# Editorial: Alternatives to traditional animal-based foods: production, characterization and comprehensive evaluation

**DOI:** 10.3389/fnut.2026.1962871

**Published:** 2026-09-23

**Authors:** Michael Rychlik, Heiko Briesen, Marius Henkel

**Affiliations:** 1 Chair of Analytical Food Chemistry, Life Science Engineering Department, School of Life Sciences, Technical University of Munich, Munich, Germany; 2 Chair of Process Systems Engineering, Life Science Engineering Department, School of Life Sciences, Technical University of Munich, Munich, Germany; 3 Professorship of Cellular Agriculture, Life Science Engineering Department, School of Life Sciences, Technical University of Munich, Munich, Germany

**Keywords:** *Alternaria*, cell-based cultivated food, cellular agriculture, mycotoxins, plant-based milk alternatives, precision fermentation, QuEChERS

Worldwide dietary changes are partly due to consumers questioning the traditional production and consumption of animal-based food for its impact on animal welfare, energy waste, loss of biodiversity, and, last but not least, being detrimental to human health. Therefore, alternatives to traditional animal-based foods are increasingly developed, marketed, and consumed.

The products include plant-based meat and milk alternatives along with products derived from cellular agriculture or tissues produced by protein-based additive manufacturing.

These alternatives reveal different properties, which confer advantages or disadvantages compared to each other and to their traditional counterparts. These aspects are elaborated in this Research Topic (RT). The collected contributions provide complementary perspectives spanning ingredient development, product characterization, process engineering, and sustainability assessment, thereby reflecting the multidisciplinary nature of next-generation food research.

The first publication in this RT deals with milk analogs produced from plant-based sources using precision fermentation (PF) (Warren et al.). This process involves genetically modified microorganisms that produce dairy proteins being fed sugar beet (SB). The lipid component of the PF milk is introduced through oilseed rape (OSR) extraction. The UK case study presented here calculates that the land footprint of the PF milks' components is only 4% of that of cow's milk. This PF milk may significantly alleviate pressure from dairy land use. However, the study considered neither the nutritional nor the safety aspects of the milk analog. Regarding nutritional value, the authors assume that adding vitamins and minerals will render the product equivalent to cow's milk. With respect to food safety, possible contaminations with mycotoxins need to be considered. In OSR, the presence of *Alternaria* toxins has been reported (1); in particular, highly hydrophobic components such as alternariol or its monomethyl ether may be transferred into the lipid phase and then introduced in the PF milk. We have previously reported on the risks of *Alternaria* toxins in plant-based milk alternatives (2) and, in this view, the resulting PF milks would need to be monitored.

Two contributions in this RT address a key challenge in developing plant-based meat alternatives: the need to simultaneously optimize nutritional quality and product structure. While one study focuses on composition and nutritional value, the other investigates the microstructural basis of texture, together providing complementary perspectives on the comprehensive evaluation of commercially available plant-based meat analogs.

Koczka et al. examined the fatty acid composition of commercially available plant-based meat substitutes and compared them with beef-based products available on the Hungarian market. Their analyses revealed substantial heterogeneity among the samples, largely driven by differences in the vegetable oils used in formulation. Plant-based products formulated with rapeseed or sunflower oil generally displayed more favorable fatty acid profiles and nutritional indices than beef products, whereas coconut oil-based formulations showed less advantageous profiles due to their higher saturated fatty acid content. The study highlights that plant-based meat substitutes cannot be treated as a nutritionally uniform category and emphasizes the importance of ingredient selection when designing products intended to provide health benefits alongside sustainability advantages.

While nutritional composition is an essential aspect of product quality, consumer acceptance of meat alternatives depends heavily on their texture and sensory properties. Pan et al. addressed this challenge by developing a novel three-dimensional characterization approach for the fibrous microstructure of plant-based meat analogs. Using micro-computed X-ray tomography combined with granulometry-based image analysis, the authors quantified fiber width, orientation, anisotropy, and structural organization in both prototype and commercial soy-based products. Their results demonstrated that extrusion temperature has a pronounced influence on fiber formation. Increasing temperatures led to progressively larger fiber structures, whereas anisotropy first increased and then decreased, indicating the existence of an optimal processing window for generating well-aligned fibrous architectures. Commercial products exhibited coarser and less anisotropic structures, likely reflecting their more complex formulations. The proposed methodology provides a valuable quantitative framework for linking processing conditions to microstructural features that ultimately determine textural performance.

Together, these latter two studies illustrate that successful plant-based meat analogs require optimization across multiple scales. Beyond replacing animal-derived ingredients, future product development must integrate nutritional design with precise structural engineering to yield products that are both health-promoting and capable of delivering the sensory characteristics expected by consumers.

While plant-based meat analogs seek to replicate the sensory and nutritional characteristics of meat using alternative raw materials, cultivated meat aims to more closely reproduce conventional meat by replacing the production process itself rather than the final product. Although cultivated meat has emerged as a promising technology with the potential to address sustainability, animal welfare, and food security challenges, its translation from laboratory research to industrial-scale manufacturing and commercial production remains a major hurdle. Addressing these engineering challenges is therefore essential for realizing the technology's full potential. Brenner et al. addressed this challenge by developing a comprehensive process model for suspension-based cultivated meat production that integrates media preparation, sequential cell expansion, downstream processing, extrusion, and supporting utilities into a single engineering framework. Rather than proposing a final production process, the model establishes a transparent reference system that enables systematic evaluation of different manufacturing scenarios and identifies technical bottlenecks before large-scale implementation. Their simulations revealed that media preparation and sterilization, cell expansion, and utility-intensive operations such as cleaning-in-place and cooling represent major constraints for process scale-up. Moreover, the model identified depleted culture medium and utility streams as critical targets for future sustainability improvements, including media recycling and waste valorization. By providing complete mass and energy balances based on explicitly stated assumptions, the framework offers a reproducible foundation for techno-economic and environmental assessments of cultivated meat production. These findings emphasize that advancing cultivated meat requires not only biological innovation but also rigorous bioprocess engineering. As the field transitions from laboratory research toward commercial manufacturing, transparent process models will be indispensable for identifying engineering priorities, comparing alternative production strategies, and guiding investments toward scalable and resource-efficient production systems.

Taken together, the studies presented in this RT highlight that the development of alternatives to conventional animal-based foods extends far beyond identifying novel ingredients. Instead, their successful implementation depends on coordinated advances across the entire value chain, from ingredient development and product characterization to scalable manufacturing and comprehensive evaluation.
